# Liver Function Tests

**Published:** 1951-04

**Authors:** G. Kemp McGowan

**Affiliations:** Chemical Pathologist, Bristol Royal Infirmary


### LIVER FUNCTION TESTS

BY

G. KEMP McGOWAN, B.A., B.M., B.Ch.
*Chemical Pathologist, Bristol Royal Infirmary*

---

The liver may be regarded as a system of branching bile ducts which terminate in numerous blind tubules, the bile canaliculi. The walls are formed by the liver cells, which are externally in contact with the capillaries of the portal venous system and the hepatic arterial system: these cells are homogeneous and show no obvious signs of differentiation or specialization. But in spite of its anatomical simplicity the liver performs a great variety of physiological functions. These are predominantly metabolic, and involve taking up substances from the blood, and liberating the same or other substances into the blood again: they include the intermediary metabolism of the three main classes of food-stuffs, and various syntheses and detoxications. In addition the liver secretes and excretes various substances to form bile.

The liver plays an indispensable part in the body economy and its importance is probably reflected in its great reserve power, for life is possible after more than four-fifths has been destroyed or thrown out of action. It also has great powers of regeneration, probably as a result of its relatively undifferentiated structure. Tests have been devised for almost every known function of the liver. They are therefore numerous, but only a few are of sufficient value and simplicity for routine use.

Many of the tests are based upon the breakdown of haemoglobin to bile pigments, which occurs in the following way. When red blood cells are broken down by the reticulo-endothelial cells, the haemoglobin is converted into " haemo-bilirubin ". This is liberated into the blood stream, carried to the liver and excreted into the bile, slightly modified, as " chole-bilirubin ". In the intestine this pigment is converted by bacterial action into stercobilin, a yellow-brown pigment which gives faeces their normal colour. Some of this is reduced still further to a colourless substance, stercobilinogen. The stercobilinogen is partially absorbed into the blood stream, and thence is almost completely re-excreted by the liver, except for a trace which appears in the urine, under the name of urobilinogen: on standing, this becomes oxidized back to stercobilin, known in urine as urobilin. Bilirubin itself does not normally appear in the faeces except in new-born infants whose intestines do not yet contain the bacterial flora necessary for converting it into stercobilin.

### Blood Bilirubin

The normal plasma bilirubin level is about 0.5 ± 0.3 mg/100 ml. When the level rises to about 2 mg/100 ml., clinical jaundice appears. Jaundice may be of three different types, haemolytic, obstructive or hepatocellular. In haemolytic jaundice, destruction of red cells results in the formation of haemobilirubin more rapidly than it can be excreted by the liver. In obstructive jaundice, there is reabsorption of cholebilirubin from the distended bile passages back into the blood. In hepatocellular jaundice which results from infective, toxic or other conditions which directly damage the liver cells, there is diminished excretion of haemobilirubin which therefore accumulates in the blood: in addition, there is commonly obstruction of the bile canaliculi by swollen liver cells, so that cholebilirubin, too, passes into the blood. Such intrahepatic obstruction may be so severe as to result in almost complete absence of bile from the intestines.

Hepatocellular damage may also be superimposed on the other forms of jaundice: in the haemolytic form due to the associated anaemia and in the obstructive form due to either biliary infection, or to the mechanical effect of a long continued obstruction.

*The Van den Bergh test* is used to investigate the bile pigments in blood. The direct test is a qualitative test to indicate whether the bilirubin present is mainly cholebilirubin (immediate reaction), haemobilirubin (delayed or negative reaction) or both (biphasic reaction). In recent years its practical value has been questioned on the grounds that if a fairly strong immediate reaction appears, it is difficult to detect a subsequent delayed reaction: and further that a biphasic result is of little diagnostic value, because it indicates liver cell damage which may be a complication of obstructive or even of haemolytic anaemia as well as the result of a primary hepatocellular jaundice. It must be admitted that the delayed reaction is of little value. But the immediate direct reaction is certainly useful, because it is nearly always absent in haemolytic jaundice, and nearly always present in obstructive or hepatocellular jaundice. The indirect test gives a quantitative estimation of the total bilirubin present, cholebilirubin and haemobilirubin. It is of unquestioned value for this purpose.

*The Icterus Index* is a simple but crude measure of the plasma bilirubin, by direct comparison with yellow colour standards. Owing to the liability of error due to other yellow pigments, it is not advisable to use it for diagnostic purposes, but it may be useful for following progress in known cases of jaundice.

### Bile in Urine

The examination of urine for bilirubin is of great value, especially when a sensitive test (*e.g.* the Harrison-Fouchet test) is used in conjunction with a less sensitive test (*e.g.* the iodine ring test): the former is used to detect very small quantities of bilirubin, the latter to indicate roughly the amount of bilirubin present. Each test reacts with both haemo- and chole-bilirubin. Haemobilirubin does not appear in the urine in more than trace quantities, however high the blood level, so that in uncomplicated haemolytic disease there will either be a reaction with the sensitive test only or none at all. Cholebilirubin, however, appears in the urine in obstructive or hepatocellular jaundice when the plasma level rises above 1 mg/100 ml.: a positive result with the sensitive test may therefore be obtained in the pre-icteric stage. There is not usually enough bilirubin in the urine to give a reaction with the less sensitive test until the plasma level reaches about 2 mg/100 ml. and clinical jaundice is present.

The testing of urine for bile salts is not a very satisfactory or useful procedure.

The urine may contain an excess of urobilin or urobilinogen as a result of increased production (haemolysis), increased absorption from the gut (constipation) or diminished re-excretion by the liver (hepatocellular disease). The amount in the urine is diminished by obstruction (biliary or intrahepatic) or by diminished absorption (diarrhoea). Thus the amount found may be the result of many opposing factors, but it is generally found to be increased in hepatocellular disease (acute or chronic), haemolytic disease, and many febrile diseases. In obstructive jaundice, the amount in urine depends on the amount of bile reaching the intestine, and the degree of associated liver damage: it is commonly increased in partial obstruction, but always disappears when obstruction is complete. In general, the testing of urine for urobilin or urobilinogen is of rather limited diagnostic value for liver disease.

### Bile in Faeces

The colour of the stools should always be noted, for in the absence of steatorrhoea it indicates roughly the amount of bilirubin reaching the intestines. This amount is high in haemolytic disease, and low in biliary obstruction, or in hepatocellular jaundice with severe intrahepatic obstruction.

### Liver Excretory Function

The main substance (other than bilirubin) which has been used for testing the excretory function of the liver is bromsulphalein. This dye is removed from the blood almost exclusively by the liver, and when a test dose is injected intravenously the rate of disappearance from the blood is taken as a measure of hepatic function. If a dose of 5 mg. per kilogram of body weight is injected, the concentration in a blood sample taken forty-five minutes later should be not more than 5 per cent of that in a sample taken five minures after the injection. The test may be made rather more sensitive if plasma concentration is also measured fifteen and thirty minutes after the injection. This test has been used extensively in America, and published results show that it is both sensitive and specific for liver cell disease, whether acute or chronic. It is, however, not suitable for use in obstructive jaundice.

### Protein Metabolism

It is believed that plasma albumen is produced by the liver, and that plasma globulins derive mainly from the cells of

TABLE I — Usual Findings in the Commoner

| | | Van den Bergh | | Urine | |
|---|---|---|---|---|---|
| | | Immediate Direct | Indirect | Bilirubin | Urobilin and Uro-bilinogen |
| Infective Hepatitis | Very Early | O to ± | N to ± | ± | N |
| | Middle .. | + | + to ++ | + | + |
| | Late .. | O to ± | N to ± | O | N |
| Cirrhosis | | O to + | N to + | O to + | + |
| Obstructive Jaundice | Partial .. | + | ± to ++ | ± to ++ | N to ± |
| | Complete .. | + | +++ | +++ | O |
| | Complicated | + | + to ++ | ± to ++ | + |
| Haemolytic Jaundice | | O to ± | ± to ++ | O to ± | N to ++ |

N=Normal level. O=Negative or absent. ±=Trace or weak positive.

the reticulo-endothelial system. In liver cell disease plasma albumen tends to fall below its normal level of 3.5–6.0 per cent., while plasma globulins tend to rise above their normal level of 1.5–3.0 per cent. The combination of a low albumen and raised globulin is very suggestive of some form of hepatitis. It is not a very sensitive test, but is usually found positive in the more chronic types of hepatitis (*e.g.* cirrhosis).

### Flocculation Tests

There is a large number of flocculation tests which depend upon abnormalities of the serum proteins. The most useful are the thymol turbidity and flocculation tests of MacLagen. The thymol turbidity test gives a normal value of less than four units. It is most commonly positive in liver cell disease, particularly in infective hepatitis (100 per cent.), less so in " serum " or " syringe " hepatitis (about 65 per cent)., and still less in cirrhosis (about 33 per cent.). It is generally negative in the hepatitis of chemical poisoning (*e.g.* arsenic), in obstructive jaundice (sometimes even when complicated by liver cell damage or infection) and in carcinoma of the liver (primary or secondary). It is occasionally positive in diseases which are

FORMS OF LIVER DISEASE AND JAUNDICE

| Faeces | Serum | | | |
|---|---|---|---|---|
| Visible Stercobilin | Alkaline Phos-phatase | Albumen | Globulin | Thymol Tests |
| N | N | N | N | N |
| N to low | + | N | N to high | ++ |
| N | N | N | N | + |
| N | N to + | Low | High | N to ++ |
| N to low | N to ++ | N | N | N |
| Low | + to ++ | N | N | N |
| N to low | N to ++ | N to low | N to high | N to + |
| N to ++ | N | N | N | N |

+=Positive. ++=Strong positive.

not primarily hepatic, *e.g.* congestive heart failures, sub-acute bacterial endocarditis, rheumatoid arthritis, infective mononucleosis and virus pneumonia: but there may of course be some liver malfunction in these conditions.

The thymol flocculation test, which is merely a second stage of the thymol turbidity test, is normally negative: when positive it has a significance similar to that of the turbidity test, but it appears to be slightly more specific for liver disease. There are many other flocculation tests used in the investigation of liver disease, the best known being the colloidal gold and cephalin cholesterol tests: but these do not possess the combined sensitivity and specificity of the thymol tests.

### CARBOHYDRATE METABOLISM

The liver plays an essential part in the metabolism of glucose, but this is not found to be significantly altered except in the more severe cases of liver disease. And as such alterations may also result from abnormalities of other organs, *e.g.* pancreas, adrenals, pituitary, thyroid, more attention has been paid to the metabolism of other sugars. Laevulose (fructose) and galactose are also taken up by the liver from the blood, but are less affected by diseases of other organs. They may be administered orally or intravenously, and the rate of liver uptake can be deduced by following either the concentration in the blood or the amounts excreted in the urine. The best test is probably the intravenous galactose tolerance test with estimations of blood galactose levels. It is specific and moderately sensitive, and as it is unaffected by biliary obstruction it may be used to assess liver cell damage in that condition: it is however rather laborious to perform.

### Lipid Metabolism

In obstructive jaundice there is usually a rise in the total blood cholesterol. On the other hand, in jaundice from liver cell disease the total cholesterol is generally normal, but there is a fall in the proportion of cholesterol esters to free cholesterol. As these changes are neither very consistent nor well correlated with the severity of liver disease the test is little used nowadays.

### Detoxication

Benzoic acid is detoxicated in the liver by combination with glycine to form hippuric acid, which is excreted in the urine. On this basis Quick developed his hippuric acid excretion test, in which the excretion of hippuric acid is measured after a test dose of sodium benzoate administered orally or, preferably, intravenously. This simple test is a fairly sensitive index of liver cell malfunction, and is quite specific provided that abno·malities of renal excretion are excluded; it is therefore advisable to carry out a simultaneous urea clearance or phenol red excretion test. The hippuric acid test can be performed in the presence of biliary obstruction.

### Miscellaneous

The serum alkaline-phosphatase level is usually increased above its normal range (3-13 units/100 ml.) in liver disease; the reason is not known. The increase is greatest in obstructive jaundice, when values of more than 40 units/100 ml. are common. In hepatocellular disease the increase is moderate, usually below 30 units/100 ml. In haemolytic jaundice the level is normal. Although the changes in phosphatase level are not constant enough to be absolutely diagnostic, in conjunction with other tests they are often of diagnostic value.

### Clinical Applications

Liver function tests are used for two main purposes: for the differential diagnosis of diseases of the liver and for assessing the degree of liver cell damage. For differential diagnosis no single test is adequate, and it is necessary to employ a " battery " of several tests. It is convenient to employ those tests which can all be carried out on a single sample of clotted blood. Those most commonly used are the Van den Bergh, the serum alkaline-phosphatase, the serum albumen and globulin levels, and the thymol turbidity and flocculation tests. In addition, the urine should be examined for bilirubin, and the stool examined macroscopically for pigment. In most cases the results of these tests, in conjunction with the clinical findings, will be enough to establish the diagnosis. The usual findings in the commoner forms of liver disease and jaundice are given in Table 1. For the diagnosis of very mild forms of hepatocellular disease when the routine tests are all normal the bromsulphalein test may be valuable.

Assessment of the degree of liver cell malfunction may be required, for instance, in following up cases of acute hepatitis to determine the degree of residual damage; in determining the degree of associated liver cell damage in diseases which are not primarily hepatocellular; or in judging the suitability of patients with liver disease (whether due to biliary obstruction or cirrhosis) for surgical treatment. When additional tests are required the bromsulphalein test is probably the best when obstruction is absent: when obstruction is present the hippuric acid excretion test is best if renal function is satisfactory, and the galactose tolerance test when it is not. These extra tests are rarely necessary, for the standard tests mentioned above give a good deal of information on such points. For instance, the thymol test is the last to become negative following infective hepatitis and is a sensitive index of incomplete resolution. In cirrhosis the serum albumen test is useful for selecting patients for lieno-renal-anastomosis, for patients with a level of less than 3 per cent. are unlikely to do well.

### SUMMARY

Of the many tests of liver function which are available only half a dozen or so are of much value in routine clinical practice. Owing to the great variety of diseases which must be distinguished in the differential diagnosis of liver disease, no single liver function test is adequate for all purposes. In most cases sufficient information can be obtained from a group of simple tests which can be performed on a single sample of serum. Occasionally one of the more elaborate tests is necessary to gain additional knowledge.

